# Fiber up‐sampling and quality assessment of tractograms – towards quantitative brain connectivity

**DOI:** 10.1002/brb3.588

**Published:** 2016-10-26

**Authors:** Stefan Sommer, Sebastian Kozerke, Erich Seifritz, Philipp Staempfli

**Affiliations:** ^1^ Institute for Biomedical Engineering, University and ETH ZurichZurichSwitzerland; ^2^ MR‐Center of the Psychiatric Hospital and the Department of Child and Adolescent PsychiatryUniversity of ZurichZurichSwitzerland; ^3^ Department of Psychiatry, Psychotherapy and PsychosomaticsHospital of PsychiatryUniversity of ZurichZurichSwitzerland

**Keywords:** diffusion, error FA, error maps, fiber up‐sampling fiber optimization, tractography

## Abstract

**Background and Purpose:**

Diffusion MRI tractography enables to investigate white matter pathways noninvasively by reconstructing estimated fiber pathways. However, such tractograms remain biased and nonquantitative. Several techniques have been proposed to reestablish the link between tractography and tissue microstructure by modeling the diffusion signal or fiber orientation distribution (FOD) with the given tractogram and optimizing each fiber or compartment contribution according to the diffusion signal or FOD. Nevertheless, deriving a reliable quantification of connectivity strength between different brain areas is still a challenge. Moreover, evaluating the quality of a tractogram and measuring the possible error sources contained in a specific reconstructed fiber bundle also remains difficult. Lastly, all of these optimization techniques fail if specific fiber populations within a tractogram are underrepresented, for example, due to algorithmic constraints, anatomical properties, fiber geometry or seeding patterns.

**Methods:**

In this work, we propose an approach which enables the inspection of the quality of a tractogram optimization by evaluating the residual error signal and its FOD representation. The automated fiber quantification (AFQ) is applied, whereby the framework is extended to reflect not only scalar diffusion metrics along a fiber bundle, but also directionally dependent FOD amplitudes along and perpendicular to the fiber direction. Furthermore, we also present an up‐sampling procedure to increase the number of streamlines of a given fiber population. The introduced error metrics and fiber up‐sampling method are tested and evaluated on single‐shell diffusion data sets of 16 healthy volunteers.

**Results and Conclusion:**

Analyzing the introduced error measures on specific fiber bundles shows a considerable improvement in applying the up‐sampling method. Additionally, the error metrics provide a useful tool to spot and identify potential error sources in tractograms.

## 1 Introduction

Diffusion magnetic resonance imaging (Le Bihan et al., 1986) is a compelling tool for probing microscopic tissue properties and diffusion tensor imaging (DTI) has become a popular model to inspect white matter architecture.

Tractography algorithms are able to reveal global fiber structures by estimating continuous streamline connections based on the local diffusion information throughout the brain (Basser, Mattiello, & LeBihan, 1994a,b). The performance of tracking algorithms has significantly improved by considering the information contained in orientation distribution functions (ODF) or fiber orientation distribution (FOD), especially in regions with complex fiber configurations (Behrens, Berg, Jbabdi, Rushworth, & Woolrich, 2007; Fillard et al., 2011; Tournier, Mori, & Leemans, 2011). However, tractograms remain biased by algorithmic‐specific parameters, that is, stopping criteria, curvature thresholds, seed point distribution, and the choice of the tracking algorithm itself, as well as partial volume effects of different fiber populations or various tissue types within the acquired data voxels. This complicates the estimation of reliable tractograms and thus the extraction of biologically meaningful connectivity measures between brain areas which are a crucial requirement for an accurate, quantitative connectome across different populations (Jbabdi & Johansen‐Berg, 2011; Jones, 2010; Jones, Knösche, & Turner, 2012). Lastly, besides validation of diffusion pipelines with dedicated phantom data mainly focusing on geometrical metrics of fiber tracts (Côté et al., 2013), there is currently no objective way to inspect the quality of tractograms in vivo, especially with respect to accurate quantification of tracking errors.

The quantification of white matter properties based on diffusion data also remains challenging. Fiber‐specific metrics are quantified by the generally unreliable fiber‐count (Jones et al., 2012) or ROI‐based approaches. The evaluation of diffusion metrics along segmented tractography bundles was introduced by (Colby et al., 2012) and (Yeatman, Dougherty, Myall, Wandell, & Feldman, 2012). The Automated Fiber Quantification (AFQ) framework allows the automatic identification and segmentation of major white matter tracts and evaluates scalar diffusion measures such as fractional anisotropy (FA) along these trajectories to quantify changes within the tract diffusion profiles among different subjects or groups (Yeatman et al., 2012). A first attempt to correct for tractography biases by estimating an actual contribution for each tract was introduced by Sherbondy et al. using a stochastic algorithm on a supercomputer architecture (Sherbondy, Dougherty, Ananthanarayanan, Modha, & Wandell, 2009; Sherbondy, Rowe, & Alexander, 2010). Another method introduced by Smith et al. is based on a nonlinear gradient descent method called spherical‐deconvolution informed filtering of tractograms (SIFT). This approach removes fibers of an initially large fiber population to improve the fit between the streamline distribution in each voxel and the fiber ODF (Smith, Tournier, Calamante, & Connelly, 2013). Thereby, a cost function describing the deviation between fiber densities and FOD lobe integrals is minimized by iteratively removing fibers. Fiber densities are calculated by incorporating the length and tangent of reconstructed fibers within a voxel and compared to the corresponding fiber ODF lobes. However, the SIFT approach requires a large amount of initial fibers to determine an optimized subset of included and excluded fiber tracts.

Its successor, SIFT 2 (Smith, Tournier, Calamante, & Connelly, 2015) reduces this requirement, as it determines an effective cross‐sectional area for each streamline, represented by a floating‐point weighting factor for each fiber, instead of a binary keeping or removing of fibers in comparison to the initial SIFT.

Pestilli, Yeatman, Rokem, Kay, & Wandell (2014) introduced a similar method, that is, linear fascicle evaluation (LiFE), which is based on the diffusion signal, predicted from the connectome, instead of the FOD. The default forward model is a degenerated tensor representing a stick with zero radial diffusivity. To deal with isotropic compartments, the signal mean is subtracted in each voxel prior to the optimization. Daducci, Dal Palu, Lemkaddem, & Thiran (2015) pursued a similar approach introducing the Convex Optimization Modeling for Microstructure Informed Tractography (COMMIT) framework, though using a more complex forward model by describing both the intracellular stick model, and the extracellular compartment by a tensor. Furthermore, gray matter and cerebrospinal fluid (CSF) are also represented with two distinct isotropic components. It is tempting to interpret the resulting fiber weights as quantitative connectivity measures between brain regions, however, the described optimization methods have their own pitfalls. For example, in voxels with poor or incorrect fiber representations due to tracking errors, noise or partial volume contaminations, compartments are typically overcompensated by increasing the weights of the few present fibers, isotropic or extracellular compartments in order to decrease the global fit error. An overview of pitfalls and open challenges is given in (Daducci, Dal Palu, Descoteaux, & Thiran, 2016).

Here, we propose a novel approach which enables the inspection of the quality and validation of a tractogram optimization such as COMMIT by evaluating FOD characteristics of the error signal along and perpendicular to fiber bundles by utilizing the AFQ framework. The quality metrics proposed allow for a better understanding of the accuracy and error sources of tractograms and help identifying regions with poorly fitted data. We further show that these metrics, combined with a newly introduced error FA, allow a better interpretation of the directional error distribution. These are important steps toward interpreting fiber weights from a tractogram optimization in a quantitative way to, for example, construct a more meaningful connectivity measure in a connectome. Furthermore, we also present a fiber up‐sampling procedure: It allows to increase the number of streamlines of a given fiber bundle, in case of, for example, underrepresentation of a certain structure due to anatomical properties, fiber geometry, seeding pattern or algorithmic constraints. Analyzing the introduced error measures on specific fiber bundles shows the benefit of using up‐sampled fiber bundles.

## 2 Materials and Methods

**Figure 1:**
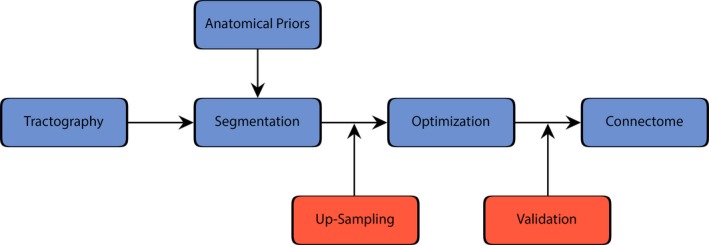
A schematic connectome pipeline is depicted including the positions for proposed up‐sampling and validation steps

The major steps of a typical connectome generation process is shown in a simplified form in Figure 1. It is crucial to perform the optimization after the segmentation and up‐sampling steps in order to avoid the partial fiber problematic discussed in (Daducci et al., 2016). In this work, in contrast to a connectome pipeline, the segmentation step is not based on cortical parcellation, but performed using the AFQ framework (AFQ: RRID:SCR_014546). This choice was motivated by the ability of the AFQ framework to reliably quantify measures along tracts.

The method section is organized as follows. First, the acquisition protocol, preprocessing steps and tractography algorithm is described. However, these parameters can easily be swapped with other protocols or tractography algorithms. Thereafter, the AFQ segmentation, fiber up‐sampling, COMMIT optimization and error quantifications, including the introduced error measures are described in more detail.

### 2.1 In‐vivo diffusion data acquisition

Diffusion MRI data were acquired on a Philips Achieva 3T TX system (Philips Healthcare, Best, the Netherlands), equipped with 80 mT/m gradients and a 32‐element receive head coil array, using a diffusion‐weighted single‐shot spin echo EPI sequence. The study was approved by the local ethics committee and meets the guidelines of the declaration of Helsinki. Written informed consent was obtained from all subjects.

Data sets from 16 healthy volunteers (age: 31.6 ± 8.6, gender: 12 male, 4 female) were acquired with the following diffusion scan parameters: TR: 11.85 s, TE: 66 ms, FOV: 220 × 220 mm^2^, with 40 contiguous slices, slice thickness: 2.3 mm, acquisition and reconstruction matrix: 96 × 96, SENSE factor: 2, partial Fourier encoding: 60%. Diffusion‐weighted images were acquired along 64 directions distributed uniformly on a half‐sphere with a b‐value of 3000 s/mm^2^ in addition to a b = 0 s/mm^2^ scan, resulting in a scan time of approximately 13 min. Additionally, 1 mm isotropic T1‐weighted structural images were recorded with a 3D MP‐RAGE sequence (FOV: 240 × 240 × 160 mm^3^, sagittal orientation, 1 × 1 × 1 mm^3^ voxel size, TR: 8.14 ms, TE: 3.7 ms, flip angle: 8°).

### 2.2 Preprocessing and tractography

For each data set, the diffusion data was corrected for eddy‐currents and subject motion by FSL: RRID:SCR_002823 (EDDY) (Jenkinson, Beckmann, Behrens, Woolrich, & Smith, 2012). The white matter mask was estimated from the T1‐weighted data set using the tissue segmentation in SPM8: RRID:SCR_007037 (www.fil.ion.ucl.ac.uk/spm) and transformed back to diffusion space using SPMs coregister function based on normalized mutual information. A Fiber Assignment by Continuous Tracking (FACT) inspired deterministic algorithm generalized to the Orientation Distribution Function (ODF) was used in the tractography step. The ODF was reconstructed using the FRACT method (Haldar & Leahy, 2013). The tracking direction was selected according to the local diffusion maximum of the ODF. Ten seeds were started in each white matter voxel, resulting in approximately 700,000 fibers per subject. The estimated white matter mask was only used for seeding purposes and was not utilized as a tractography stopping criterion.

### 2.3 Fiber segmentation and up‐sampling

The segmentation of the tractograms was performed using the AFQ framework (Yeatman et al., 2012), which is based on a waypoint ROI procedure as described in (Wakana et al., 2007). Additionally, a refinement step was applied, which compares each candidate fiber to tract probability maps (Hua et al., 2008). To avoid conflicting start and endpoints of fibers running through the two ROIs of the target fiber structure, a flip was performed on all tracts which first passed through the second ROI, resulting in consistent fiber alignment in each bundle. These segmentation steps resulted in the selection of 20 major white matter fiber tracts (Yeatman et al., 2012) out of all white matter fibers contained in the whole‐brain tractogram (18 bundles as described in (Yeatman et al., 2012), and two additional tracts as defined in the online version: https://github.com/jyeatman/AFQ).

Next, to increase the number of fibers of potentially underrepresented fiber populations in the different AFQ segmented bundles, for example, due to tractography algorithm biases, the following method was applied: The segmented fibers were equidistantly resampled using 80 interpolation points per fiber and principal component analysis (PCA) was applied to all classified and resampled fibers (Parker et al., 2013). The space was truncated to the first 80 dimensions (from the 240 point descriptors), whereby more than 99% of the explained variance was still captured. In the PCA space, for each bundle separately, new fibers were randomly generated according to the point distribution of the transformed fibers, assuming a bundle‐specific multivariate Gaussian distribution. The newly generated fibers were transformed back by inverting the linear PCA transformation.

In a further step, potential outliers were identified based on the calculation of a population‐mean fiber, that is, the mean value of all corresponding resampled points of the initial fibers within one fiber bundle. The distance of each randomly generated fiber to the original population‐mean fiber was derived by summing up the distances to the nearest points on the mean fiber. New fibers were only accepted if the distance‐threshold to the initial population was met. This threshold was set to the maximum fiber distance of all fibers within the initial population relative to its mean fiber. Newly generated tracts leaving the white‐matter mask were also rejected. Based on these fiber population up‐sampling steps, additional 10,000 fibers per bundle were generated for each data set.

Finally, the up‐sampled fibers were again segmented using the AFQ framework to apply the same classification criteria to the newly generated fibers as to the initial tractogram. Around 75% of the up‐sampled fibers were successfully classified and therefore kept for the further analysis. With the procedure described above, a total of four tractography sets were generated:

**Table:** 

AFQ	Classified AFQ fibers based on the initial tractogram
AFQUP	AFQ set combined with the up‐sampled AFQ fibers
WB	Initial whole‐brain tractogram
WBUP	WB combined with the up‐sampled AFQ fibers

### 2.4 Fiber optimization, optimized tractogram

The optimization of the different tractogram sets was performed using the COMMIT framework (Daducci et al., 2015) by applying the Stick‐Zeppelin‐Ball model (Panagiotaki et al., 2012) for modeling the fiber signal. The intracellular stick model was generated with a longitudinal diffusivity of *d*_∥_ = 1.7 × 10^−3 ^mm^2^/s. In addition, in each voxel, a hindered contribution was included for every unique FOD peak using the Zeppelin model assuming a perpendicular diffusivity *d*_⊥_ = 0.5 × 10^−3 ^mm^2^/s and longitudinal diffusivity *d*_∥_ = 1.7 × 10^−3 ^mm^2^/s. Lastly, two isotropic compartments accounting for partial volume with gray matter and cerebrospinal fluid were modeled with diffusivity $d\in \left\{1.7,3.0\right\}\times 10^{-3}\;{\text{mm}}^{2}/s$. The nondiffusion weighted b = 0 image was used to normalize the diffusion data. The convex optimization problem of the following form

$$\underset{x\geq 0}{\text{argmin}}\|Ax-y{\|}_{2}^{2}$$

where *y* is the vector containing the normalized diffusion signal, *A* is the linear operator or dictionary and *x* is the vector of the contributions, was solved using a forward‐backward, fast iterative shrinkage‐threshold algorithm (https://github.com/daducci/COMMIT), resulting in a solution $\tilde{x}$. Stopping criteria for the optimization were either a maximum number of 500 iterations or a minimum relative change of the objective function of 1e‐4.

### 2.5 Error quantification

In addition to the normalized root mean square error (NRMSE) of the optimization fit, an actual signal estimator $\hat{s}$ was calculated using $A\tilde{x}$, by reverting the b = 0 normalization. To further examine the differences and similarities between this signal estimator $\hat{s}$ and the acquired diffusion data *s*, a directional error FOD of the signal estimator $\hat{s}$ and the original diffusion data *s* was calculated. Remaining signal contributions from under‐ or overrepresented fibers are assumed to remain in the error signal. The FOD for the diffusion signal estimator was reconstructed by applying the constrained spherical deconvolution (Tournier, Calamante, & Connelly, 2007) to the error signal, which is defined by the element wise difference between the measured and estimated diffusion signals:

$$s_{\mathrm{err}}^{i}=\sqrt{{\left(s^{i}-{\hat{s}}^{i}\right)}^{2}}$$

In order to use a meaningful deconvolution kernel and to be comparable to the FOD derived from the measured signal *s*, the response function was not re‐estimated on the error signal; instead the fiber response from *s* was used. A maximum spherical harmonics order of *l*_max_ = 8 was used. Furthermore, a traditional tensor fit of the signal error *s*_err_ was derived in order to calculate the fractional anisotropy (FA) of *s*_err_.

To quantify the different error measures along the segmented and optimized AFQ fiber bundles, we extended the tract profile generation of the AFQ framework. In (Yeatman et al., 2012), the locations of the used waypoint ROIs from the segmentation step (2.3) isolate the central trajectories of the fascicles. Next, different scalar diffusion measures (FA, RD, etc.) are evaluated along the central portion of the fiber bundle by clipping and resampling each fiber according to the main segment between the ROIs. Bundle properties are then summarized at each node by taking a weighted average according to the Mahalanobis distance of each fiber tract core as described in (Yeatman et al., 2012).

**Figure 2:**
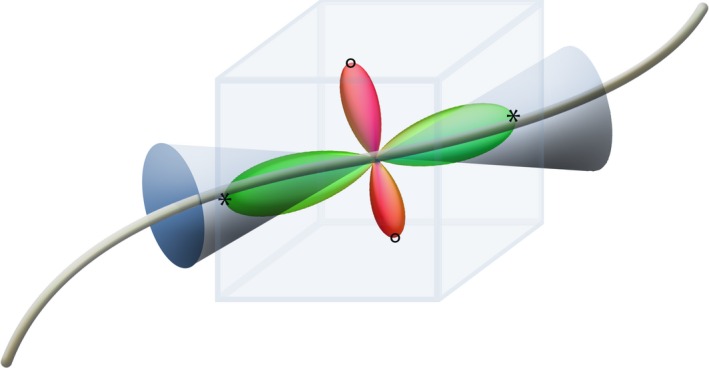
Schematics showing the fiber orientation distribution (FOD) evaluation along a fiber tract: longitudinal maxima are marked by stars (within the cone), perpendicular maxima are marked with circles (outside of the cone)

In this work, instead of investigating traditional scalar diffusion quantities as proposed in the AFQ framework, we examined scalar measures such as the fit NRMSE and the introduced error FA along the segmented AFQ tracts. Furthermore, the three‐dimensional error FOD was also evaluated by calculating longitudinal and perpendicular error FOD amplitudes for each segmented AFQ fiber. These measures depend on the fiber directionality and are not scalar maps. The maximum peak‐amplitude along a fiber tract is defined by the maximum FOD amplitude in a cone around the fiber orientation with an opening angle of *π*/6. The maximum peak‐amplitude perpendicular to the fiber is the maximum of all sampling points outside this cone (Figure 2).

For every tractogram set (*n *= 4), following parameters were analyzed along each of the 20 segmented fiber bundles: NRMSE, error FOD along, error FOD perpendicular, and error FA. These measures were tested for statistical significance between the initial and up‐sampled tractogram sets and were corrected for multiple comparison, using the nonparametric permutation test implemented in FSL (Winkler, Ridgway, Webster, Smith, & Nichols, 2014). The number of permutations were set to 5000 with a significance level of *p *< .05.

Furthermore, the up‐sampling method was also compared with an increase of seed points during the tractography step. Therefore, the number of seed points was increased incrementally up to a factor of eight in a single subject. The resulting tractogram sets were segmented using the AFQ framework and either optimized or up‐sampled and optimized for the comparison. The up‐sampled tractogram sets were also segmented a second time prior to the optimization.

## 3 Results

**Figure 3:**
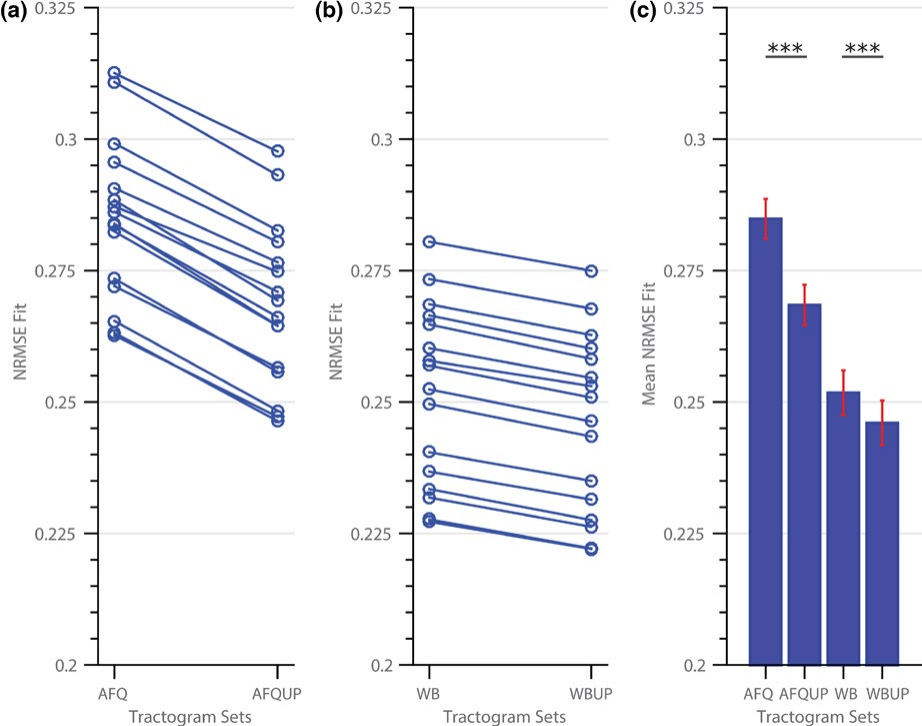
Optimization results showing the mean normalized root mean square error (NRMSE) for each subject between (a) automated fiber quantification (AFQ) and AFQUP, and (b) WB and WBUP; (c) group average for the four tractogram sets, the error bars depict one standard error

In Figure 3, the mean NRMSE of all four tractogram sets are shown for every subject (*N *= 16) after the optimization with the COMMIT framework. The error in the up‐sampled populations (AFQUP and WBUP) is decreased compared to the initial sets (AFQ and WB) for each subject, and comparison at the group level shows a highly significant decrease in the mean NRMSE between AFQ and AFQUP and between WB and WBUP (paired samples, *p *< .001). Furthermore, the whole‐brain tractograms (WB and WBUP) also showed lower errors compared to the AFQ and AFQUP.

**Figure 4:**
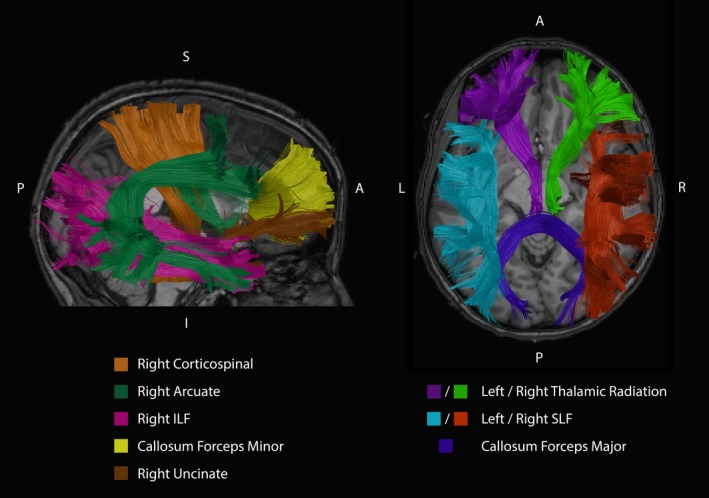
A selection of the discussed segmented fiber bundles of a single representative subject are shown in different colors. In the sagittal view, the right Corticospinal Tract, right Arcuate Fasciculus, right Inferior Longitudinal Fasciculus (ILF), the Callosum Forceps Minor, and the right Uncinate Fasciculus are illustrated. The axial slice depicts the left and right Thalamic Radiation, left and right Superior Longitudinal Fasciculus (SLF) and the Callosum Forceps Major

**Figure 5:**
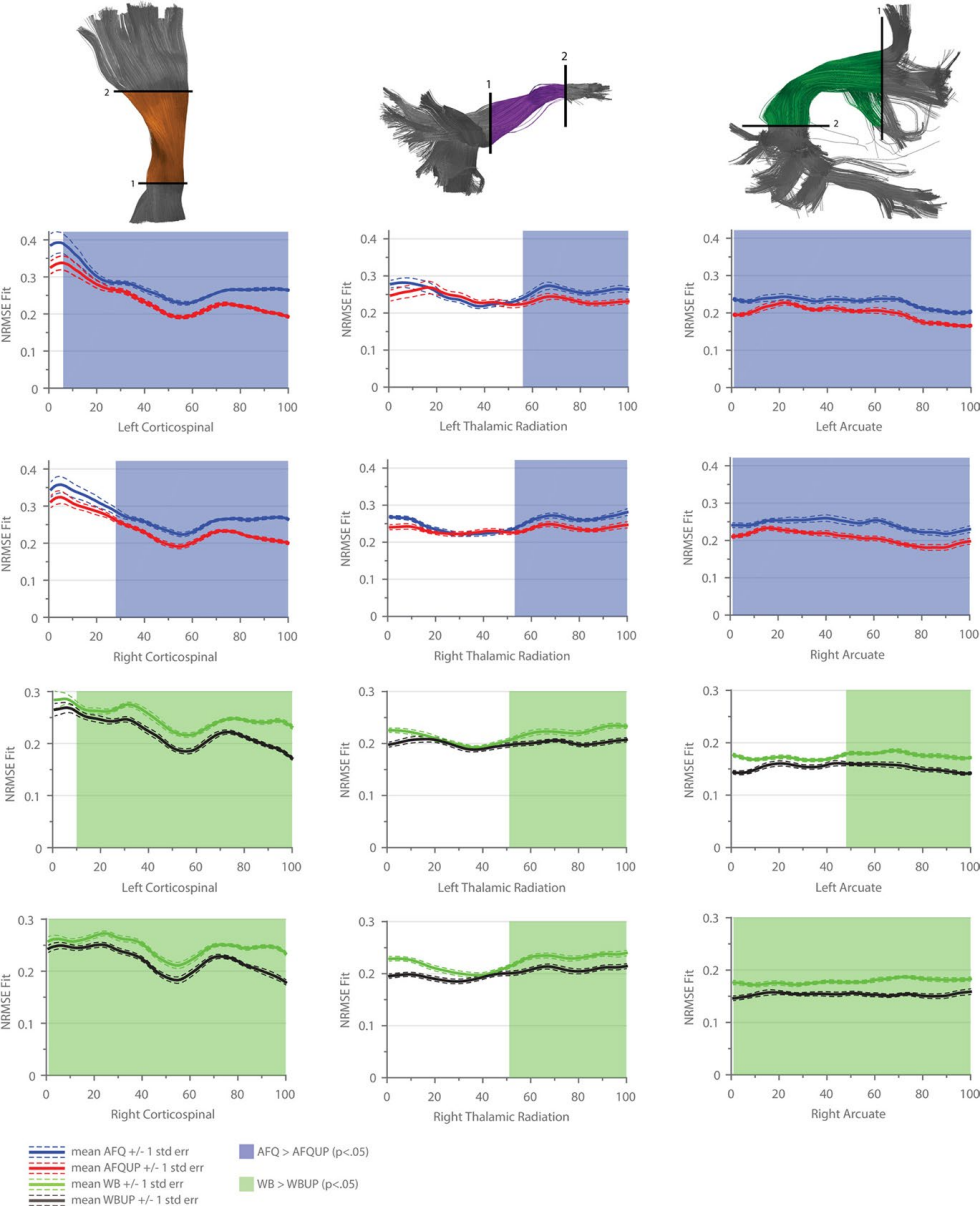
Mean normalized root mean square error (NRMSE) of the optimization over all subjects for the left and right Corticospinal Tract, Thalamic Radiation, and Arcuate Fasciculus. Subplots 1–6 shows the mean NRMSE of the automated fiber quantification (AFQ) fiber set (blue,) and of the up‐sampled tractogram set AFQUP (red). The dashed red and blue lines indicate one standard error. Subplots 7–12 shows the mean NRMSE of the WB fiber set (green), and of the WBUP‐tractogram set (black). The dashed green and black lines indicate one standard error. Areas with a significant error reduction (according to FSL's randomize, *p *< .05) in AFQUP compared to AFQ are overlaid in transparent blue (AFQ > AFQUP) or green (WB > WBUP), respectively

**Figure 6:**
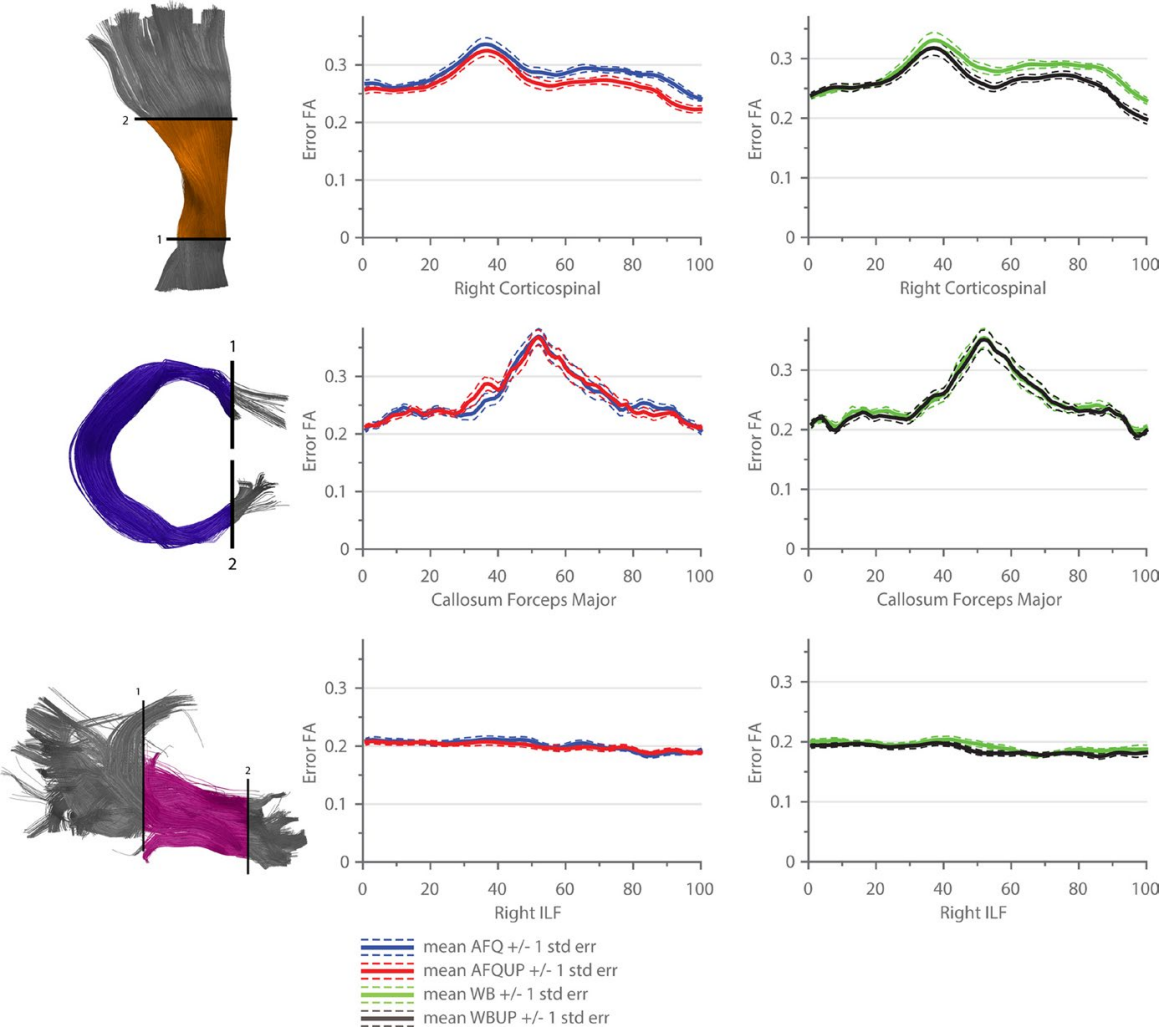
The fractional anisotropy (FA) of the error signal is shown along three selected bundles (Corticospinal Tract, Callosum Forceps Major, and Inferior Longitudinal Fasciculus). The mean error FA over all subjects derived from the initial optimized, nonup‐sampled sets are depicted in blue (automated fiber quantification [AFQ]) and green (WB), the error FA derived from the up‐sampled sets are displayed in red (AFQUP) and black (WBUP). The dashed lines indicate one standard error

**Figure 7:**
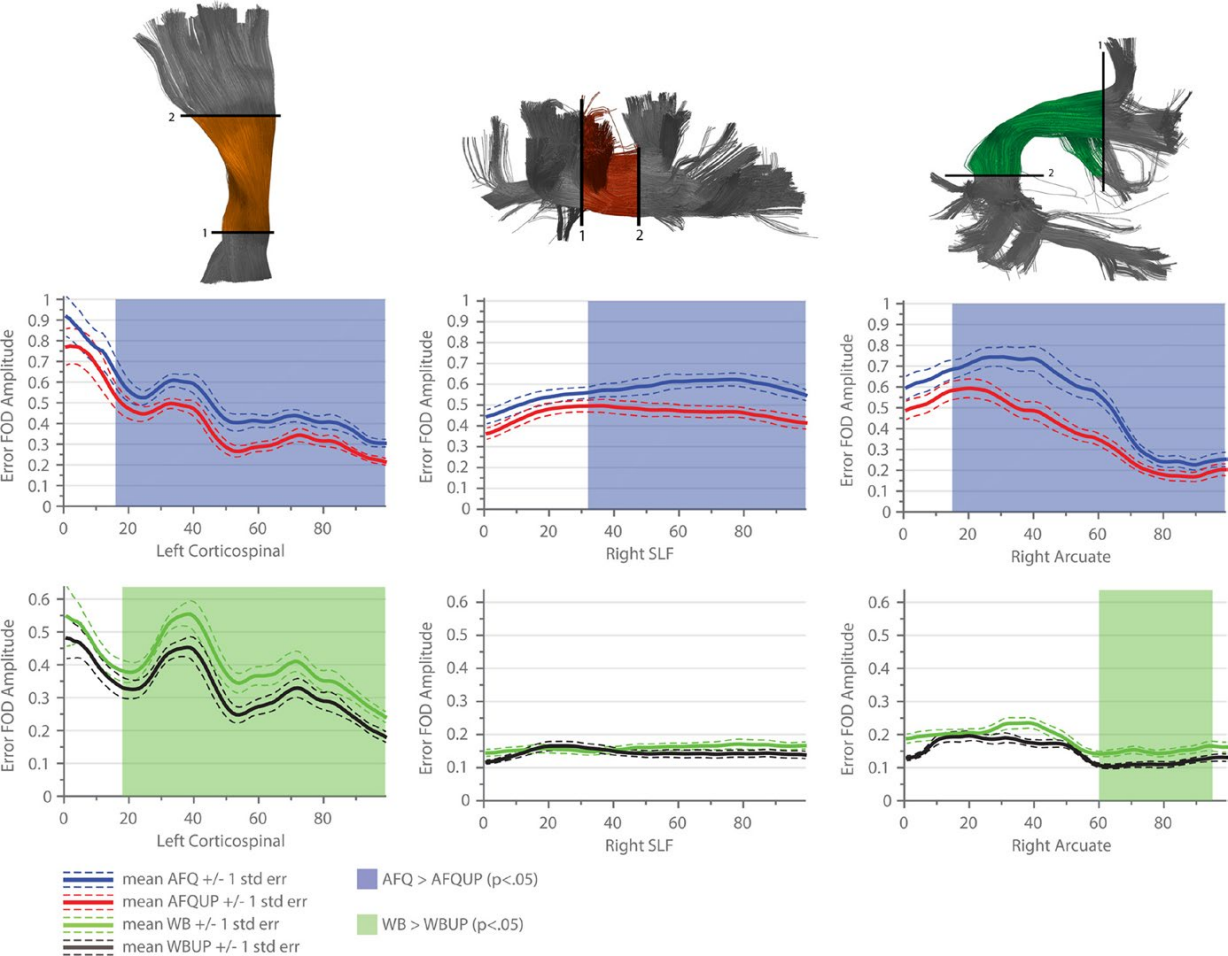
Longitudinal error fiber orientation distribution (FOD) peak amplitudes along three representative bundles are shown (Left Corticospinal, Right Superior Longitudinal Fasciculus and Right Arcuate Fasciculus). The mean across all subjects using the initial tractograms are shown in blue (automated fiber quantification [AFQ]) and green (WB), the mean longitudinal FOD error in the up‐sampled sets are depicted in red (AFQUP) and black (WBUP). The dashed lines indicate one standard error, statistically significant regions (*p *< .05) are highlighted with transparent surfaces (blue: AFQ > AFQUP, green: WB > WBUP)

**Figure 8:**
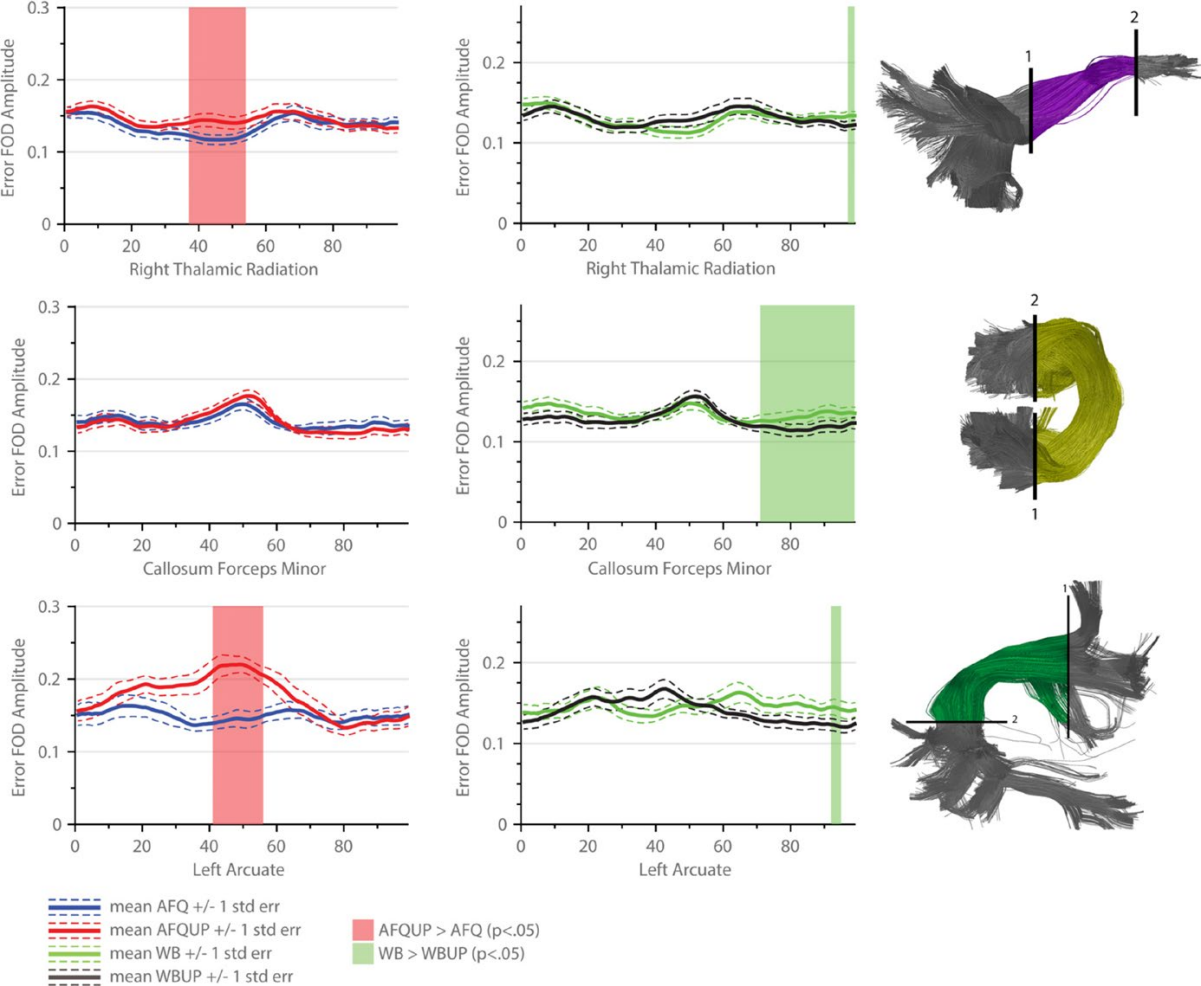
Perpendicular error fiber orientation distribution (FOD) peak amplitudes across the selected automated fiber quantification (AFQ) bundles (Right Thalamic Radiation, Callosum Forceps Minor, Left Arcuate Fasciculus): the mean over all subjects using the initial tractograms are shown in blue (AFQ) and green (WB). The perpendicular error FOD peak amplitudes in the up‐sampled sets are drawn in red (AFQUP) and black (WBUP). The dashed lines indicate one standard error statistically significant regions (*p *< .05) are highlighted with transparent surfaces (red: AFQUP > AFQ, green: WB > WBUP)

The different segmented AFQ fiber bundles that are discussed in further detail in the following sections are illustrated in Figure 4. Figures 5, 6, 7, 8 show the tract profile of the NRMSE, error FA, longitudinal and perpendicular FOD error in selected bundles to illustrate different distributions of the error signal and performance of the up‐sampling method.

Figure 5 shows the NRMSE along three major bundles (left and right hemisphere) in the four tractograms sets (AFQ, AFQUP, WB, WBUP). The colored section of the depicted bundles describe the core of the bundle, whereas the x‐axis in the subplots shows the 100 parameterized points between ROI 1 and ROI 2. In Figures 5, 6, 7, 8, the ROIs are marked with 1 and 2 to emphasize the start and end region of the parameterization.

The lower error in the up‐sampled tractograms (AFQUP, red line, WBUP, black line) compared to AFQ and WB (blue, green line) achieved a better fit compared to the initial sets (AFQ, WB). In most parts, the fit error significantly decreased (*p *< .05) after multiple comparison correction using FSL's randomize. Regions of statistical significance are highlighted with a transparent overlay in the color of the tractogram set with a higher value (e.g., blue for AFQ).

The FA of the error signal gives further insight into the optimization results. In Figure 6, three different types of error FA behavior are shown as an example. The Corticospinal Tract showed a statistically significant reduction of the error FA in the up‐sampled populations (AFQUP vs. AFQ and WBUP vs. WB), which is desirable in order to reduce a directional bias in the residual diffusion signal. Nevertheless, structural tendencies along the bundle are still visible, especially in the second quarter of the bundle, where the error FA is clearly increased in all of the tractogram sets. The error FA in the Callosum Forceps Major could not be reduced by applying the up‐sampling method, and especially in the middle part of the bundle, directional biases in the residual diffusion signal remain clearly visible. In contrast, the Inferior Longitudinal Fasciculus (ILF) revealed a relatively isotropic error signal, expressed by low FA values, and no distinct structure in the error FA, that is, no directional bias in the residual diffusion signal along the bundle was observed.

In Figure 7, the longitudinal FOD error is evaluated along the distinct fiber bundles. The Corticospinal Tract showed a significantly (*p *< .05) reduced longitudinal error in both up‐sampled sets compared to the initial tractograms. In the Superior Longitudinal Fasciculus (SLF), the up‐sampling reduced the error in the AFQ population (AFQ vs. AFQUP,). The longitudinal error was already low in the WB tractogram set for the SLF, and could not be further reduced in a statistically significant manner by up‐sampling the bundle (WBUP). The Arcuate Fasciculus showed a similar behavior, whereas the up‐sampling significantly reduced the longitudinal error in the AFQ cases. Additionally, in the WB sets, the up‐sampling still significantly reduced the longitudinal error in the temporal part of the bundle (WBUP) but the overall difference is drastically reduced.

Figure 8 depicts the perpendicular FOD error in the segmented fiber bundles of the right Thalamic Radiation, Callosum Forceps Minor and the left Arcuate Fasciculus. For the AFQ case, the up‐sampled sets showed a significantly higher error in the Thalamic Radiation and the Arcuate Fasciculus in some parts, even though the overall mean fit error (NRMSE) was reduced. If all the fibers are taken into account (WB, WBUP), the up‐sampled population (WBUP) does not show a significant increase of the perpendicular error anymore.

**Figure 9:**
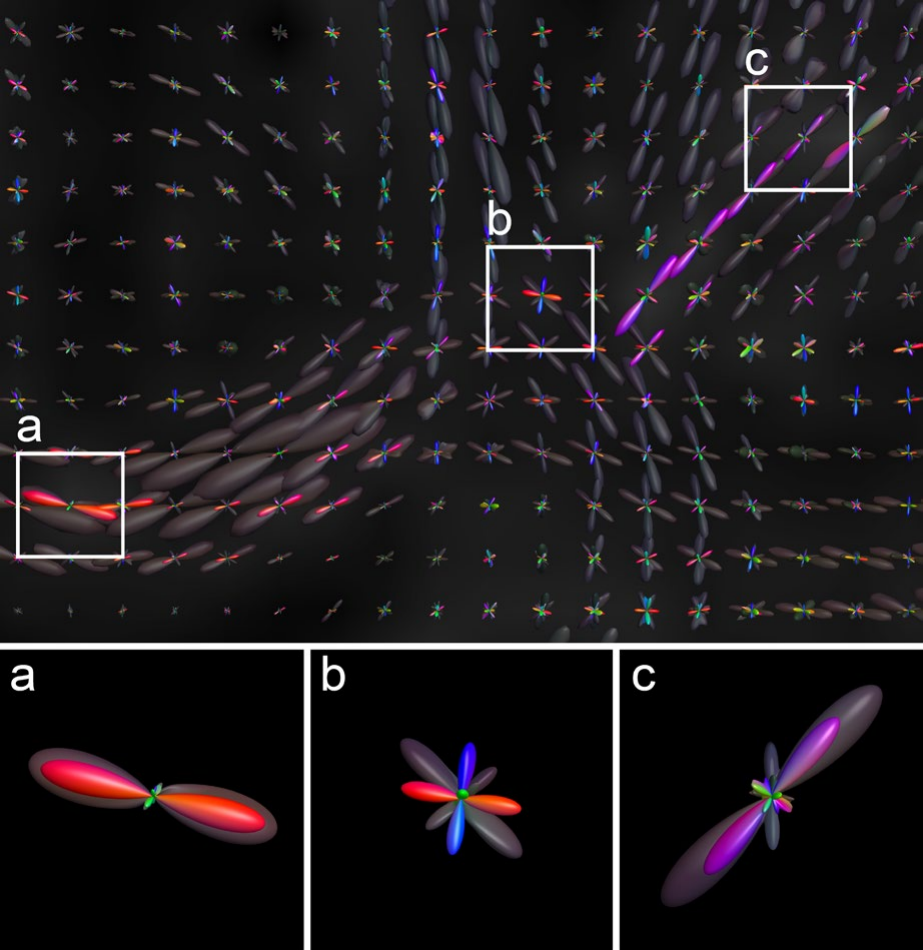
A coronal section through the Corona Radiata is shown in a single subject, whereby the signal fiber orientation distribution (FOD)s derived from the measured diffusion signal are depicted in transparent gray, the error FODs derived from the WB set are overlaid in color. Three different voxels are highlighted where the error is significantly larger compared to the other voxels

Figure 9 shows a coronal cross section through the Corona Radiata of a single subject. The reconstructed FODs from the measured diffusion signal are depicted in gray, with the colored error FODs derived from the WB set shown on top. Most voxels exhibit a small error FOD compared to the signal FOD, implicating a good agreement between the signal estimator from the optimization and the measured signal. Nevertheless, in some voxels, the error FOD is relatively large compared to the signal FOD. Three of those voxels are highlighted in a, b and c.

**Figure 10:**
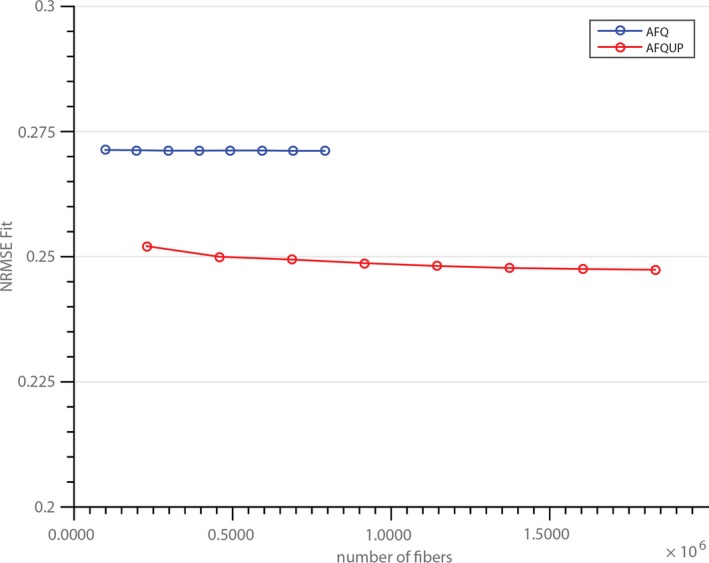
Different number of seed voxels and the resulting mean normalized root mean square error (NRMSE) for the automated fiber quantification (AFQ) and AFQUP tractogram sets are shown

Figure 10 depicts the comparison between increasing the number of seed points during the tractography and up‐sampling the segmented fiber bundles in a single subject. Each tractogram set is plotted with the number of fibers on the x‐axis in order to compare the same number of fibers. The up‐sampling method clearly outperforms the increase in seed points, whereby the largest improvement is achieved by the first up‐sampling step.

## 4 Discussion

We have introduced a tool to investigate the quality of a tractogram by further inspecting the directionally dependent error signal between the signal prediction and the measured diffusion signal along reconstructed fiber bundles. Additionally, we presented a method to up‐sample a given fiber population in order to achieve better optimization results, that is, a decreased fit error.

The overall mean fit error averaged over all the white‐matter voxels and all subjects showed only small, but nevertheless significant changes comparing the initial (AFQ, WB) with the up‐sampled (AFQUP, WBUP) fiber tractograms. These small changes at the group level could be attributed to large intersubject variability; however, the up‐sampled sets achieved a reduced fit error in each single subject (Figure 3a and b). The improved signal fit achieved by the up‐sampling method was highly statistically significant for both the AFQ vs. AFQUP and WB vs. WBUP tractogram sets. Further inspection of the NRMSE along the major segmented fiber bundles showed high similarity between the matched left and right structures. However, differences were found between various structures, for example, the superior part of the Corticospinal Tract was highly improved by the up‐sampling method, whereas the frontal part of the Thalamic Radiation was mostly unaffected by the up‐sampling procedure. Variable performance of the up‐sampling method across structures might be caused by the quality of the initial bundle representation and also by voxels surrounding these bundles. Systemic errors were expected and observed in the AFQ tractogram sets (AFQ, AFQUP) due to the fact that many fibers are not covered by the 20 major bundles and therefore excluded from the optimization. The signal of crossing, nonsegmented structures are missing in regions with high NRMSE in the AFQ and the AFQUP tractograms. While the AFQUP set showed a reduced error in almost all structures compared to the AFQ set, a compensation of nonsegmented crossing structures remains unachievable by merely up‐sampling the segmented bundles without the introduction of missing crossing structures. Segmenting the AFQ bundles introduces an additional source of error due to predefined ROIs and registration steps during the AFQ bundle classification. Fibers, which pass through the distinct bundles but, for example, not through the two ROIs are consequently unclassified, and therefore missed in the optimization. In the whole‐brain sets (WB and WBUP, Figure 5) no fiber populations were purposely omitted, and therefore a much more homogeneous NRMSE distribution was found in the brain.

In a next step, we further explored the error distribution across the diffusion directions in each voxel. Therefore, the error FA was calculated and evaluated to reveal potential anisotropy in the error signal (Figure 6). In voxels with a good fit, a low anisotropy is expected, that is, a homogeneous distribution of the error across all diffusion directions. In comparison to the NRMSE, the error FA appears to be a sensitive measure for recognizing badly represented regions, even if all the fibers are taken into account (WB, WBUP). A bad fiber representation or an inaccurate forward model can cause a high error FA as, for example, observed in the middle section of the corpus callosum and parts of the Corticospinal Tract. These bundle segments also have a high signal FA, which might indicate that the chosen forward model underperforms in high FA voxels. Despite the clear distinction of high error FA regions in the graphs, it is rather difficult to define an accurate baseline for the residual error signal in order to distinguish structurally related residuals from pure noise. An accurate signal‐to‐noise ratio estimation of the diffusion signal would be needed which is typically also spatially varying due to multiple acceleration methods.

Complex tissue architecture of crossing fiber populations within a single voxel cannot be fully modeled by tensor based metrics, therefore the FOD of the error signal was also evaluated along and perpendicular to the major fiber bundles (Figures 7 and 8). By disentangling the error signal into a perpendicular and longitudinal error, the exact source of the tractogram error can be observed. Poorly represented structures can therefore be discriminated from over‐ or underestimated crossing structures. The longitudinal error in the Superior Longitudinal Fasciculi and in the Arcuate Fasciculi differs strongly in the initial populations (AFQ, WB), which is most plausibly caused by segmentation difficulties. However, in case of the Arcuate Fasciculus, applying the up‐sampling method in the WB set further significantly reduced the longitudinal error. By further investigating the perpendicular error, a significantly higher error in the AFQUP set was identified for the first time. In the WB sets, this effect diminishes and can therefore be explained by missing fiber populations not embodied in the segmented AFQ bundles.

These fiber‐dependent directional measures combined with the error FA enable to detect and distinguish possible error sources, namely bad fiber bundle representation, missing crossing structures or a poor forward‐model fit.

Missing crossing structures in the AFQ sets can be found for example, in the lower part of the Corticospinal Tract. The Cerebellar Peduncles, are passing superior to the first Corticospinal ROI and might be the reason for an increased error FA and NRMSE. Another example is along the middle part of the Arcuate Fasciculus next to the superior region of the Corona Radiata. Besides The Superior Longitudinal Fasciculus, which is also included in the major AFQ bundles, the Posterior Vertical Arcuate and the Vertical Occipital Fasciculus also populate this area and are not segmented, using the AFQ framework.

The provided tools allow an extensive inspection of tractograms and their optimization by exploring bundle‐specific and directionally dependent error measures. To the author's knowledge, this is the first study that facilitates a deepened insight into the remaining local errors induced by the tractogram or the optimization procedure itself. This step is crucial in order to get a better understanding of the actual goodness of fit of the tractogram.

In Figure 9, different cases of error FODs are highlighted. In voxel a), the directionality of the error FOD matches the initial FOD. Most certainly, some of the passing fibers were under or over‐estimated in that particular voxel. The error FOD in voxel b) shows a completely different characteristic as the initial FOD. The geometry of the reconstructed fibers do not match the measured diffusion signal. The third case (voxel c) is a combination of both cases, where the local weighting deviates from the diffusion signal and also the error FOD peaks are slightly tilted. Other voxels show very small error FODs and some spurious peaks do occur, whereby the assumption of an underlying fiber response was violated. Nevertheless, the amplitudes of these error FODs are very small and will not influence the resulting along‐tract analysis.

A possibility to mitigate the fiber assumption of the kernel function would be to apply a model‐free Funk‐Radon‐Transformation to the error diffusion signal instead of deconvolving the error signal with a fiber response function. The resulting error orientation distribution function (ODF) would not suffer from spurious peaks.

The ultimate goal of using streamline weights to quantify connectivity strength between cortical regions is still not achievable if the tractogram or the optimization is flawed. The fiber‐dependent error estimation could also be used to estimate a confidence of the resulting fiber weights aiming to verify the integrity of a quantitative connectivity matrix between cortical regions. Additionally, the difficulty of omitting fibers due to any segmentation and its influence on to the optimization was shown. In the given framework, the segmentation method can easily be exchanged with a segmentation, ,for example, based on a cortical parcellation. Thereby, the up‐sampling can also be applied to more fiber bundles. However, other segmentation methods, for example, based on a cortical parcellation scheme also suffer from unclassified fibers which cannot be included in the tractogram optimization step.

The introduced fiber up‐sampling method improved the tractogram representation, hence led to a significantly superior optimization expressed by a reduced NRMSE. Even though, the introduced approach did not enhance the optimization in every single structure, impairment caused by the fiber up‐sampling was not observed. Additionally, the up‐sampling was performed per bundle, whereby a bundle segmentation is a necessary prerequisite.

This requirement might be eliminable, if a different sampling strategy is applied to randomly draw samples in the PCA space. The assumption of a multivariate Gaussian distribution is no longer valid in a whole‐brain fiber population and would lead to many implausible up‐sampled fibers.

In Figure 10, the fiber up‐sampling is compared with increasing the number of seed voxels during tractography. Even an eight‐fold increase of fibers did not improve the optimization result substantially. However, the up‐sampling is not only computationally less intensive, it also introduces new fibers with different features, a larger spatial extent, and therefore novel trajectories and the up‐sampled fibers do differ substantially from the initial population. These new fibers contribute significantly to a more optimal tractogram set. Similar effects were reported by (Takemura, Caiafa, Wandell, & Pestilli, 2016) while different algorithm parameters were introduced instead of only increasing the number of seed points. The presented framework also enables the comparison of different tractogram sets from various sources and allows a more extensive inspection of each tractogram and its strength and weaknesses without defining an explicit gold standard.

For a reliable fiber quantification, it is crucial to eliminate error sources such as a bad fit due to wrong choice of the forward model, poor tractogram representation caused by the choice of tracking algorithm and tractography parameters or an overcompensation in voxels with a low number of streamlines. As discussed in (Smith et al., 2015), FOD lobes of voxels containing very little streamlines compared to the actual measured fiber density will result in assigning high weights to those fibers in order to reduce the error in that particular voxel, but introducing biases in all the other voxels traversed by those fibers. Similarly, the COMMIT model will assign higher volume fractions to extracellular compartments in order to compensate for missing streamlines representing the intracellular compartment. The presented up‐sampling procedure can help to limit voxels containing a low number of streamlines, especially in cases where a higher track density cannot be achieved by simply increasing the number of seed voxels. With respect to global tractography algorithms, an increase of fibers is typically computationally very expensive, whereas the described up‐sampling method is very efficient. The introduced error measures such as the error FA or the error FOD itself could also be fed back into the tractography process to influence the placement of new seed voxels or adjust tractography parameters in order to achieve a more representative tractogram. In this study, only single shell diffusion data was used for the optimization, whereas the COMMIT framework clearly benefits from multiple shells, for example, in order to distinguish between different isotropic compartments.

## Conflicts of Interest

None declared.
